# *Neisseria gonorrhoeae* infection in female sex workers in an STI clinic in Nairobi, Kenya

**DOI:** 10.1371/journal.pone.0263531

**Published:** 2022-02-25

**Authors:** Amina Abdullahi, Samson Muuo Nzou, Gideon Kikuvi, Matilu Mwau

**Affiliations:** 1 Centre for Infectious and Parasitic Diseases Control Research, Kenya Medical Research Institute (KEMRI), Busia, Kenya; 2 Centre for Microbiology Research, Kenya Medical Research Institute (KEMRI), Nairobi, Kenya; 3 School of Public Health, Jomo Kenyatta University of Agriculture and Technology (JKUAT), Nairobi, Kenya; Massachusetts General Hospital, UNITED STATES

## Abstract

**Background:**

Gonorrhea caused by *Neisseria gonorrhoeae* is the second most prevalent curable sexually transmitted infection worldwide. Female Sex Workers (FSWs) are at a higher risk of contracting gonorrhea due to their risky sexual behaviors like inconsistent condom use and multiple sexual partners. We determined the prevalence and risk factors associated with gonorrhea and its antimicrobial susceptibility pattern among symptomatic FSWs attending Sexual Workers Outreach Program (SWOP) city clinic in Nairobi, Kenya.

**Methods:**

Using convenience sampling, we recruited 379 female sex workers from SWOP City clinic in Nairobi County. We administered a semi-structured questionnaire to collect data on socio-demographics and behavioral risk factors associated with gonorrhea. We also conducted three focus groups. Two endocervical swabs were collected from each participant by the attending physician for the laboratory identification of *Neisseria gonorrhoeae*. An antimicrobial susceptibility test was performed using the disc diffusion method.

**Results:**

Twenty-four out of 379 (6.3%) participants tested positive for gonorrhea by PCR. The significant risk factors associated with gonorrhea were having multiple sexual partners in the previous two weeks, primary education, and being in the age group of 38–49 years (p < 0.05).

From the qualitative data, sex work disclosure, and difficulty in engaging protected sex with their partner, and unprotected sex with their clients due to more money from the client, PREP, and alcohol use made the female sex workers vulnerable to gonorrhea exposure and or risky sexual behavior. The culture-positive sample result yielded complete (100%) resistance to all the antimicrobials used.

**Conclusion:**

*Neisseria gonorrhoeae* infection is prevalent among symptomatic FSWs in Nairobi. Multiple sexual partners, being in age group 38–49 years and having primary education were the factors associated with gonorrhea among the study participants. Based on our identification of a highly resistant isolate, we strongly recommend increasing capacity for culture-based diagnosis and susceptibility testing.

## Introduction

*Neisseria gonorrhoeae* is the etiological agent of gonorrhea. Every day, over one million sexually transmitted infections (STIs) occur worldwide [1]. Gonorrhea is an STI that remains a major public health concern. It is the second most curable STI in the globe and causes morbidity and economic consequences [2]. In 2016, 87 million new cases occurred among adolescents and adults aged 15–49 years worldwide, with the highest incidence in Sub-Saharan Africa [1,3]. Sub-Saharan Africa has an estimated STI incidence of 240/1000, with 21.1 million annual incidences of *Neisseria gonorrhoeae* infections [4]. STIs affect low-income countries, with prevalence estimates higher in women [5]. Symptoms include discharge and pain with urination for urethral infections, while pharyngeal and rectal infections are usually asymptomatic. Gonorrhea is a major cause of the pelvic inflammatory disease (PID) in women that leads to severe reproductive health complications. In Kenya, previous studies reported a prevalence range of 0% to 6% [6–8]. Female Sex Workers (FSWs) are at a higher risk of contracting STIs due to their risky behaviors, including unprotected sex, multiple sex partners, and alcoholism [5,9]. FSWs are a vulnerable group with a high risk of acquiring STIs [8].

In Kenya, STI treatment is based on evidence of symptoms suggestive of gonococcal infection. According to the ministry of health guidelines in Kenya, the antibiotics that would be prescribed for vaginitis, cervicitis, and PID include Clotrimazole, Metronidazole 400mg RX, Norfloxacin 800mg, and Doxycycline 100mg.

The treatment of *Neisseria gonorrhoeae* infection is complicated by its ability to develop resistance to antimicrobials. Internationally, resistant strains have been documented, with the majority of gonococcal treatment failures to oral cephalosporins reported from European and Asian countries [3]. In recent years, *Neisseria gonorrhoeae* has developed a decreased susceptibility to ceftriaxone and cefixime. *Neisseria gonorrhoeae* has also been found to be resistant to extended-spectrum cephalosporins (ESCs) [10]. Cefixime failures have been reported in Japan [11]. Only one resistant strain with an MIC of > 0.25 ug/ml (MIC = 0.5 ug/ml) was reported globally. Recently, the first ceftriaxone-resistant gonococcal strain H041 with a MIC of ceftriaxone of 2ug/ml was isolated from a female commercial sex worker in Tokyo [12]. Although the studies are few, in Kenya, quinolone resistance in western Kenya, coastal, and Nairobi has been documented [13].

The Kenya National Guidelines for Prevention, Management, and Control of STIs 2018 report indicated that there is little information on STI incidences in Kenya, particularly among FSWs. Thus, our study determined the prevalence, risk factors, and antimicrobial susceptibility profile of *Neisseria gonorrhoeae* among FSWs in the SWOP City Clinic.

## Materials and methods

### Study design and population

The study adopted a cross-sectional design using both quantitative and qualitative methods. A sample size of 346 participants was determined to be sufficient, but in the end, 379 participants were recruited by convenience sampling. Eligibility criteria included being between the ages of 18 and 49, having STI symptoms, and not having received antibiotic treatment in the previous two weeks.

### Data collection

Semi-structured questionnaires administered by the first author were used to collect socio-demographic factors (age, marital status, level of education) and behavioral factors (number of sexual partners, condom use, HIV status, pelvic pain, and history of STI in the past one year) and symptoms (vaginal discharge, itchy vulva, foul smell, and painful urination. Three Focus Group Discussions (FGDs) with eight participants each were conducted. The recruitment of the FGD participants was done using the list of participants from the quantitative study. From the list, we randomly called participants by phone and enrolled those who consented. Using an FGD guide that described a case of a FSW named Jennifer who was married to John and had one child. She was in sex work for 10 years. She hid her sex work from her husband, and he knew she was a supermarket attendant. Jennifer engaged in risky sexual behavior. In the above scenario, the participants were asked questions using the guide. The FGDs lasted an hour each. By the time we did the third FGD, we had reached saturation point (no new theme was emerging).The FGDs were conducted in English and Kiswahili, and all the participants were able to speak and understand at least one of the languages. The FGDs were conducted by a trained FGD facilitator and the first author. At the beginning of each FGD, the participants were reminded of confidentiality. Interesting themes that emerged from previous group discussions were further explored in the subsequent ones till saturation was reached. All the discussions were audio-recorded, translated, and later transcribed. All the transcripts were coded and analyzed using NVIVO8.Two endo-cervical swabs were collected from each participant by a trained clinician. The swabs were transported to the laboratory using Stuart transport medium (HiMedia Laboratories Pvt. Ltd., India).

### Laboratory analysis

#### Isolation and identification of *Neisseria gonorrhoeae*

The endocervical swab from each participant was smeared on a glass slide for microscopy and cultured. After microscopy, the swabs were inoculated onto GC medium plates and incubated in 5% CO2 at 37°C. After 18–24 hours, they were examined for typical growth. If there was growth, oxidase and catalase tests were done to confirm *Neisseria gonorrhoeae*. *Neisseria gonorrhoeae* is oxidase positive and ferments glucose but not maltose, sucrose, or lactose.

Cultures showing no typical growth were re-incubated for an additional 24 hours and examined again for growth. Plates with no typical growth or oxidase negative were discarded after 72 hours of incubation.

#### PCR

A direct PCR was performed in the KEMRI-NUITM laboratory to confirm the presence of *Neisseria gonorrhoeae*. The direct PCR was done using the mighty amp reagents and the simpliprep machine. Specifically designed primers, forward-CGGCAGCATTCAATTTGTTAAAGC and reverse-CGCCATTTTTGTA, which were to yield a PCR product of 162 base pairs each, were used [14]. The thermal cycle conditions for the simpliprep machine were: 94°C, 60 sec (denaturation), 59°C, 60 secs (annealing), 72°C, 26 sec/max 429bp (1 min. kbp), (elongation), 72°C, 10 minutes (extended elongation). When the reaction was over, the samples were loaded on gel electrophoresis, stained, and viewed under ultraviolet light (UV).

The antimicrobial susceptibility testing was done by disk diffusion using 13 antimicrobials from Liofilchem Company (Ciprofloxacin (CIP,30μg), Ceftriaxone (CRO,30μg), Cefepime (FEP,25μg), Cefotaxime (CTX,30μg), Cefuroxime (CXM,30μg), Cefazolin (KZ,30μg), Ceftazidime (CAZ,30μg), Cefoxitin (FOX,30μg), Tetracycline (TE,30μg), Ampicillin (AMP,10μg**),** Amikacin (AK,30μg), Gentamycin (CN,30μg) and Amoxillin (AUG,30μg**).**The antimicrobial discs were inoculated onto a culture of *Neisseria gonorrhoeae* and incubated for 24–48 hours. The zone of inhibition was measured and interpreted using the CLSI 2016 guidelines.

### Data analysis

Quantitative data was entered and analyzed in Stata/MP Version 13 for Windows. Basic descriptive statistics were used to describe the frequencies and percentages of the variables and the prevalence of *Neisseria gonorrhoeae* infection among the study participants. The level of education variable was categorized using the Kenyan education system of 8:4:4. This consists of 8 years of primary education, 4 years of secondary education, and 4 years of university education. In the analysis, we pooled no formal education and primary education (≤ primary) and secondary and above-secondary education (≥ secondary).The number of sexual partners’ variable was analyzed using the median. The median of the number of sexual partners in the previous two weeks was calculated and categorized into ≤ the median and ˃ the median in the analysis. Univariate logistic regression was used to determine the association between the independent variables and the outcome. All the variables that were significant (p <0.05) in the univariate analysis were fitted into a multiple regression model to better understand their association with gonorrhea. For the qualitative data, all the interviews were audio-recorded, transcribed, and coded through the creation of categories and themes using NVIVO8. Data was presented through the insertion of verbatim quotations to augment or contrast the quantitative data. The level of significance was set at P <0.05.

### Ethical approvals

This study was conducted in accordance with the Declaration of Helsinki. Ethical approval was sought from the KNH-UON Ethical Review Committee (Ref. No. KNH/ERC/R/138). Written consent was provided by all the participants.

## Results

### Demographic and socio-behavioral characteristics of the study participants

Three hundred and seventy-nine participants were enrolled. The median age of the participants was 29 years (IQR 10). The median number of sexual partners in the past 2 weeks was 29.The Majority (93.7%) of the participants was single, and 73.9% had secondary education. A total of 16 (4.2%) study participants had never or occasionally used condoms, while 51 (13.5%) were HIV positive. A total of 241(63.6%) study participants reported having more than 29 sexual partners in the past two weeks. Thirty-five (9.2%) participants reported having an STI in the previous year (Table 1).

**Table 1:** Demographic and socio behavioral characteristic of the study participants.

Socio-demographics		Category	Frequency	Percent
Age group	18–27		159	42.0
	28–37		162	42.7
	38–49		58	15.3
Marital status	Single		355	93.7
	Married		24	6.3
Level of Education	No formal education		1	0.3
	Primary		70	18.5
	Secondary		280	73.9
	Above secondary		28	7.3
Number of sexual partners in the previous 2 weeks	≤29		138	36.4
	˃29		241	63.6
HIV status	Negative		328	86.5
	Positive		51	13.5
Condom use	Always		363	95.8
	Never/occasionally		16	4.2
History of STI in the previous year	No		344	90.8
	Yes		35	9.2

### Prevalence and clinical symptoms

The prevalence of *Neisseria gonorrhoeae* infection was 6.3%. The highest prevalence (13.8%) was in the age group of 38–49 years. Eight out of 58 participants in the age group of 38–49 years tested positive for gonorrhea.

Three hundred and forty-seven (91.7%) of the study participants had abnormal vaginal discharge, while 275 (72.6%) had pelvic pain. The other clinical symptoms were: painful urination 28 (7.4%), foul smell 82 (21.6%), and itchy vulva 48(12.7%). We used logistic regression to analyze the clinical symptoms associated with gonorrhea. None of the clinical symptoms were significantly associated with *Neisseria gonorrhoeae* infection (Table 2).

**Table 2:** Clinical presentations of the study participants.

Symptom	Frequency (%) n = 379	N. gonorrhoeae Negative (%) n = 355	N. gonorrhoeae Positive (%) n = 24	p-value
**Abnormal discharge**				
Yes	347(91.7)	323(90.98)	24(100)	
No	32(8.3)	32(9.02)	0(0)	-
**Painful urination**				
Yes	28(7.4)	27(96.4)	1(3.6)	0.54
No	351(92.6)	328(93.4)	23(6.6)	
**Foul smell**				
Yes	82(21.6)	81(98.8)	1	
No	297 (78.4)	274(92.3)	23(7.7)	0.06
**Itchy vulva**				
Yes	48(12.7)	46(95.8)	2(4.2)	0.50
No	331(87.3)	309(93.4)	22(6.6)	
**Pelvic pain**				
Yes	275 (72.6)	256(93.1)	19(6.9)	0.46
No	104(27.4)	99(95.2)	5(4.8)	

### Risk factors associated with *Neisseria gonorrhoeae* infection

From the univariate analysis, being in the age group 38–49 years compared to 18–27 years, having more than 29 sexual partners in the previous two weeks and having primary education compared to secondary education were the risk factors associated with *Neisseria gonorrhoeae* infection. Being in age groups: 18–27 and 28–37 years, level of education (Secondary and beyond), HIV status, condom use, history of STI in the past one year, the number of sexual partners in the previous two weeks and presence of STIs symptoms (pelvic pain, itchy vulva, painful urination, discharge, and foul smell) were not significant risk factors (Table 3).

**Table 3:** Univariate logistic regression for the risk factors associated with *Neisseria gonorrhoeae* infection among the study participants, n = 376.

	*Neisseria gonorrhoeae* infection			OR (95% CI)	p value
	Positive (n = 24) (%)	Negative (n = 355) (%)			
**Age group**					
18–27 (159)	7(4.4)	152(96.2)		Referent	
28-37(162)	9(5.6)	153(94.4)		1.28(0.46–3.5 1)	0.64
38-49(58)	8(13.8)	50(86.2)		3.47(1.20–10.06)	**0.02**
**Level of education**					
≤ Primary (71)	10(14.1)	61(85.9)		3.17(1.34–7.47)	**0.01**
≥Secondary(308)	14(4.5)	294(95.5)		Referent	
**Number of sexual partners in the previous 2 weeks**					
≤29(138)	3(2.2)	135(97.8)		Referent	
˃29(241)	21(8.7)	220(91.3)		4.29(1.26–14.67)	0.02
**HIV status**					
Positive (51)	3(5.9)	48(94.1)		0.91(0.26–3.18)	0.89
Negative (328**)**	21(6.4)	311(93.6)		Referent	
**Presence of pelvic pain**					
Yes (255)	19(7.5)		236(92.5)	1.47(0.57–3.8)	0.43
No (124)	5(4.0)		119(96)	Referent	

OR: Odds Ratio.

CI: Confidence interval.

Female sex workers aged 38–49 years were more likely to get *Neisseria gonorrhoeae* infection compared to the younger age groups (OR 3.47, 95% CI 1.20–10.06). Also, female sex workers with primary education were more likely to get *Neisseria gonorrhoeae* infection compared to those with secondary education (OR 3.17, 95% CI 1.34–7.47). The odds of getting *Neisseria gonorrhoeae* infection was four times higher among the FSWs who had more than 29 sexual partners in the previous two weeks compared to those who had 29 and less sexual partners (Table 3). All the variables that were significant in the univariate analysis were also significant in the multiple logistic regression model (Table 4).We managed to isolate only one sample by culture and it showed 100% resistance to all the antimicrobials used.

**Table 4:** Multiple logistic regression analysis for the risk factors associated with *Neisseria gonorrhoeae* infection among the study participants, n = 379.

Variables	OR	95% CI	P value
**Age**			
18–27 (159)	referent		
28-37(162)	1.02	.35–2.97	0.97
38-49(58)	2.80	.89–8.84	0.08
**Level of education**			
≤ Primary	0.28	.11-.71	0.01
≥Secondary	referent		
**Number of sexual partners in the previous 2 weeks**			
≤29(138)	referent		
˃29(241)	5.09	1.46–17.74	0.01

AOR: Odds Ratio.

CI: Confidence interval.

### Antimicrobial susceptibility testing

In our study, antimicrobial susceptibility testing of the isolated bacterium (n = 1) was performed against 13 antimicrobials from Liofilchem company using the disk diffusion method. The result showed complete (100%) resistance to all the antimicrobials.

### Qualitative data

According to most of the respondents, couples who are at risk of contracting sexually transmitted diseases (STDs) such as gonorrhea should be honest with one another, especially about their occupation and the likelihood of having relationships with multiple partners that could expose them to sexually transmitted diseases (**Box 1**). Most of the respondents highlighted that it would be good for couples to be faithful to one another to avoid contracting STDs, as it might lead to dire consequences such as divorce. In the event that one of them contracts some of these diseases, most of the respondents felt it would be prudent for the couple to seek treatment together to avoid recurrent exposure.

**Box 1: Honesty among couples**

*“Jenifer should have told her husband because he is her husband*. *She should have told him the truth so that they see how they can live well together*…*”***FGD, Nairobi**

In seeking to understand if couples who are at risk of contracting STDs should use protective modern family planning methods such as condoms, it emerged that, according to most of the respondents, it would be difficult for couples to agree to this as this would encourage unfaithfulness in the marriage.

During the focus group discussions with respondents, it was clear that most of the respondents were cognizant of pre-exposure prophylaxis (PREP) and its role as they indicated that it is used for prevention of human immunodeficiency virus (HIV) (**Box 2**), especially among individuals who are at high risk of contracting it due to their risky sexual behaviors.

**Box 2: Role of PREP**

*“The role of PREP is to prevent a person from contracting HIV*.*”***FGD, Nairobi**

In the view of most of the respondents, the main driver for engaging in risky sexual behaviors among individuals is the use of PREP, since some people have the perception that it will not only protect them from HIV but also from STDs (**Box 3**), and alcohol consumption, which can inhibit a person’s judgment on the use of protective methods such as condoms. Some also indicated that due to the current hard economic times, some people might engage in risky sexual behaviors to get money for basic needs.

**Box 3: Drivers to practicing risky sexual behaviors**

*“Jenifer is practicing risky sexual behaviors because she feels she is very safe*, *she is using PREP yes*, *she won’t get any disease*…*”***FGD, Nairobi**

## Discussion

Gonorrhea is a global public health problem. In 2012, the World Health Organization reported *Neisseria gonorrhoeae* infection as the second most prevalent STI globally, and it causes morbidity and economic loss. In this study, the prevalence of *Neisseria gonorrhoeae* infection was 6.3%. This is comparable to what was previously reported in another study on the patterns and risk factors of STDs in women attending an STD referral clinic in Nairobi, 6% [7], 8.3% Yunnan, China [15], 9.1% Isfahan, Iran [16], However, this rate was higher than 1.1% in Melbourne [17], 3.3% in Hawassa, Ethiopia [18], and other studies in Kenya included 1.1% in Nairobi [19], 1.8% in Mombasa [8], Kenya, In contrast to this, the 6.3% prevalence reported in this study is lower than the 14.1% reported in India [20]. This shows the continued burden of *Neisseria gonorrhoeae* infection. These varying prevalence rates could be due to differences in the study population, distribution of the sociodemographic risk factors, different study sites, sampling, and laboratory techniques employed by the different studies. The present study was done in a well-established STI clinic that provides counseling, testing, and treatment, and outreach programs to female sex workers so that they could be aware of the risk factors associated with STIs. Having multiple partners in the previous two weeks had a significant statistical association with *Neisseria gonorrhoeae* infection. The odds of getting *Neisseria gonorrhoeae* infection increased with an increase in the number of sexual partners. Those who reported more than 29 sexual partners in the past two weeks were four times more likely to get gonorrhea compared to their counterparts with ≤ 29 sexual partners (OR 4.29,95% CI 1.26–14.67,p = 0.02).

This finding is consistent with a study that was done in Ghana that reported females who engaged in multiple sexual partners had a significant association with *Neisseria gonorrhoeae* infection compared to those who didn’t [21]. This is because multiple sexual partners may be associated with high-risk sexual behaviors such as unprotected sex due to the amount of money offered and alcoholism. The study also found *Neisseria gonorrhoeae* infection was more prevalent among the participants with primary education (14.3%) (p = 0.01). This is comparable to a similar study done in Hawassa, Ethiopia that found a lower prevalence of *Neisseria gonorrhoeae* infection in female sex workers with secondary and above-secondary education [18]. Another study in Yunan, China also found that female sex workers with higher education levels were less likely to be infected with *Neisseria gonorrhoeae* [15]. This could be explained by the fact that women with higher education may be in a better position to read and understand better educational information [22]. The qualitative data analysis showed that it was difficult to disclose sex work to the spouse since it may have led to a breakup. It also showed that it was difficult to use condoms with their spouse since it brought mistrust into the relationship. The FGD participants noted the drivers of risky sexual behaviors are: PREP, alcohol, and more money from the sexual partner. None of the reported clinical symptoms were significantly associated with *Neisseria gonorrhoeae* infection. Although all the study participants reported symptoms consistent with *Neisseria gonorrhoeae* infection, 93.7% did not test positive for gonorrhea. This is because most STI symptoms are non-specific. The antimicrobial profile of the isolate showed complete resistance to all the antimicrobials, including ciprofloxacin and ceftriaxone that are in the current STI treatment algorithm in Kenya. In addition, a four-year study conducted at SWOP City Clinic reported that *Neisseria gonorrhoeae* antimicrobial resistance to Ciprofloxacin had increased over the years. However, contrary to the present study findings, they found 100% susceptibility to Cefixime, Ceftriaxone, and Spectinomycin. This shows an increasing rate of resistance of *Neisseria gonorrhoeae* to antimicrobials. Another study in coastal Kenya reported 71% resistance to ciprofloxacin [23]. The finding from the present study shows possible multidrug-resistant gonorrhea circulating among FSWs in Nairobi, which is of concern.

## Study limitations

Our study had several limitations. The main one was we obtained our samples from an STI clinic and that the reported prevalence is for symptomatic FSWs only. We recruited only the adult FSWs in the reproductive age group (18–49). We isolated only one sample by culture, hence, the resistance finding is based on only one sample. The study did not test for other STIs.

## Conclusion

The present study shows *Neisseria gonorrhoeae* is prevalent among FSWs in Nairobi. Being in age group 38–49 years and having primary education and having more than 29 sexual partners in the previous two weeks were the important risk factors for *Neisseria gonorrhoeae* infection.Based on our identification of a highly resistant isolate, we strongly recommend increasing capacity for culture-based diagnosis and susceptibility testing.

## Supporting information

S1 File Questionnaire. (DOCX)
