# Supplementary material for: Neisseria gonorrhoeae infection in female sex workers in an STI clinic in Nairobi, Kenya
Source: PLoS One. 2022 Feb 25;17(2):e0263531. doi: 10.1371/journal.pone.0263531 (PMC8880920; doi:10.1371/journal.pone.0263531)
Supplement: S1 File — (DOCX) [file pone.0263531.s001.docx]

**APPENDIX III:** **QUESTIONNAIRE: ENGLISH AND KISWAHILI**

The prevalence and the risk factors of Neisseria gonorrhoeae infection and its antimicrobial susceptibility profile among symptomatic female sex workers attending swop city clinic in Nairobi.

**Personal Details (Socio-demographic characteristics)**

Maelezo ya binafsi

Date………………………….

Tarehe

Study Identification number…………………… Age (years) ………………………

Nambari ya utafiti Umri

Gender: Male ( ) Female ( )

Jinsia Kiume Kike

Level of education

Kiwango cha elimu

- Primary(Msingi)
- Secondary(Upili)
- Above secondary(elimu ya juu)

Marital status (Hali ya ndoa)

- Single(hajaolewa)
- Married(ameolewa)

**Behavioral characteristics**

HIV status(hali ya ukimwi): ................................................................

The number of sexual partners in the past two weeks……………….

idadi ya washirika wa ngono wiki mbili zilizopita

Condom use(matumizi ya kondomu)

- Always(kila wakati)
- Never or occasionally(kamwe/mara kwa mara)

History of STI (in the last year)

Ugonjwa wa zinaa mwaka uliopita

- No(La)
- Yes(Specify)(ndio-bainisha)

**Symptom category**

Dalili

- Asymptomatic(bila dalili)
- Urogenital symptoms(dalili)

Other symptoms presented (dalili zingine)………………………………

Name of person completing the form (jina la mwenye anajaza fomu): ………………………... …………………………….................................

Signature (Sahihi) …………………………..

Date (Tarehe) ………………..... Time(wakati) ………………..............
